# Supplementary figures and images for: Comparative Cytological and Transcriptome Analyses of Anther Development in Nsa Cytoplasmic Male Sterile (1258A) and Maintainer Lines in Brassica napus Produced by Distant Hybridization
Source: Int J Mol Sci. 2022 Feb 11;23(4):2004. doi: 10.3390/ijms23042004 (PMC8879398; doi:10.3390/ijms23042004)

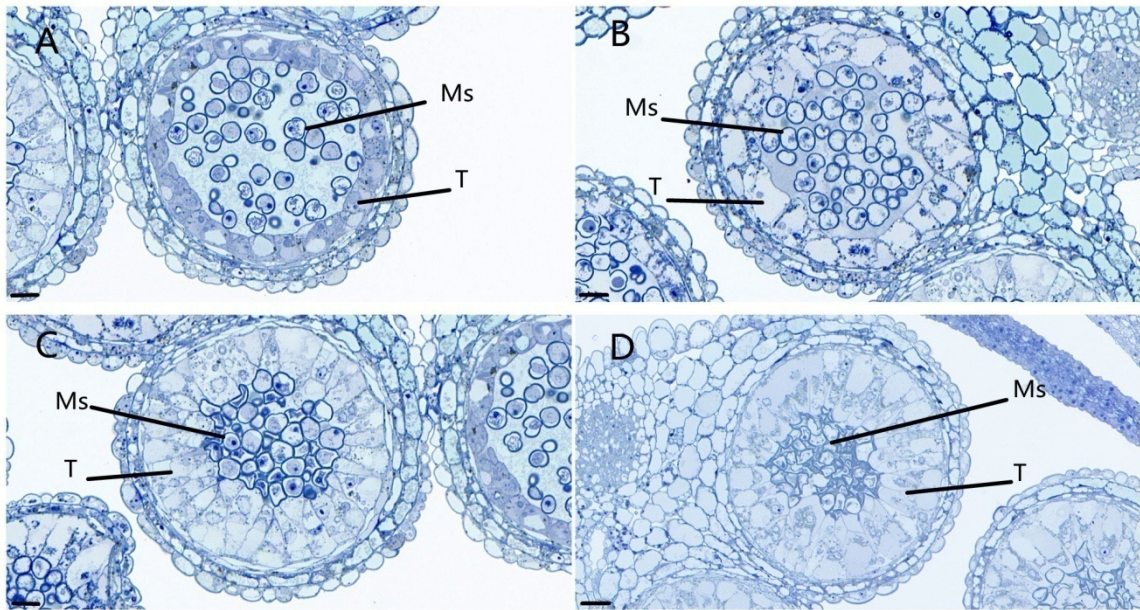

Figure S1 Tapetum cell expansion extrusion of microspores  
T,tapetum; Ms,microspore; Bar=20  $\mu$  m.

Supplement: Supplementary file 1 [file ijms-23-02004-s001.zip › Figure S1 Tapetum cell expansion extrusion of microspores.pdf]

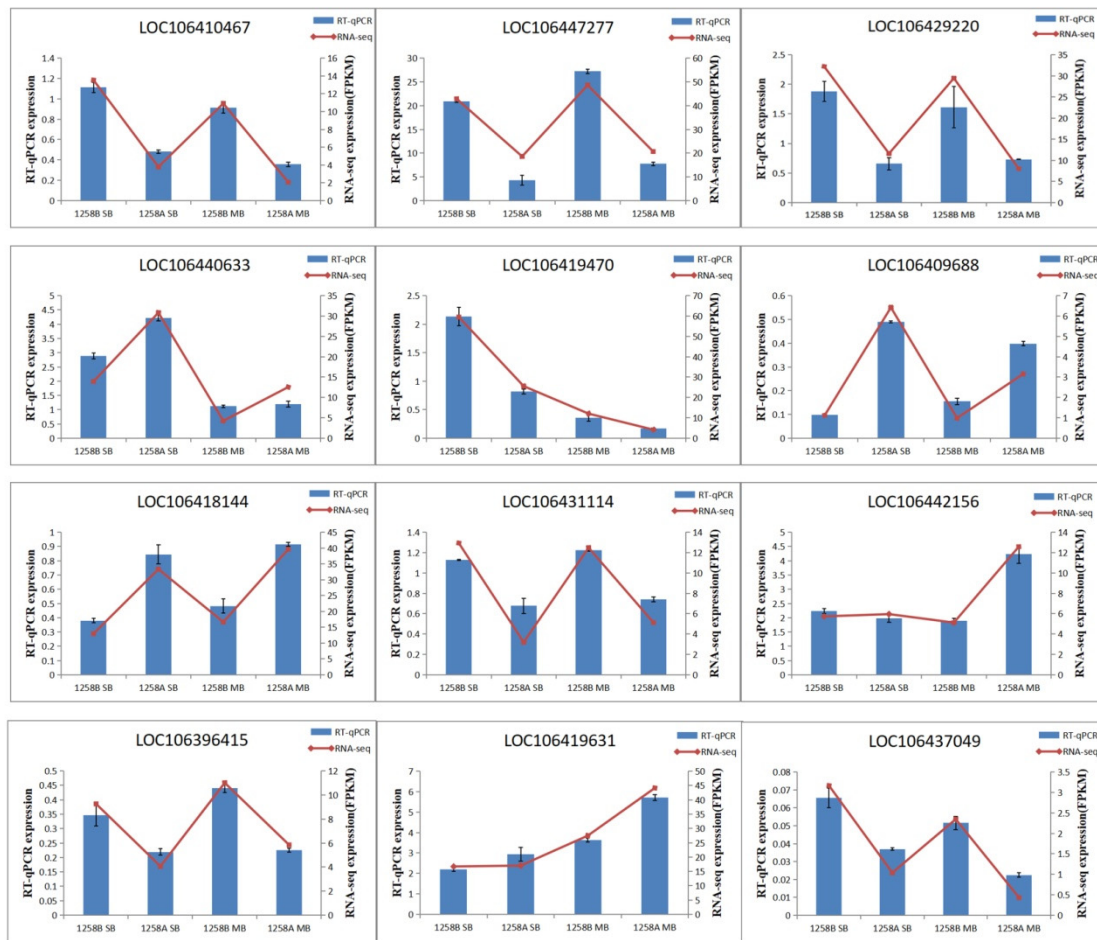

Figure S2 RT-qPCR verification results of 12 genes.

Supplement: Supplementary file 1 [file ijms-23-02004-s001.zip › Figure S2 RT-qPCR verification results of 12 genes.pdf]

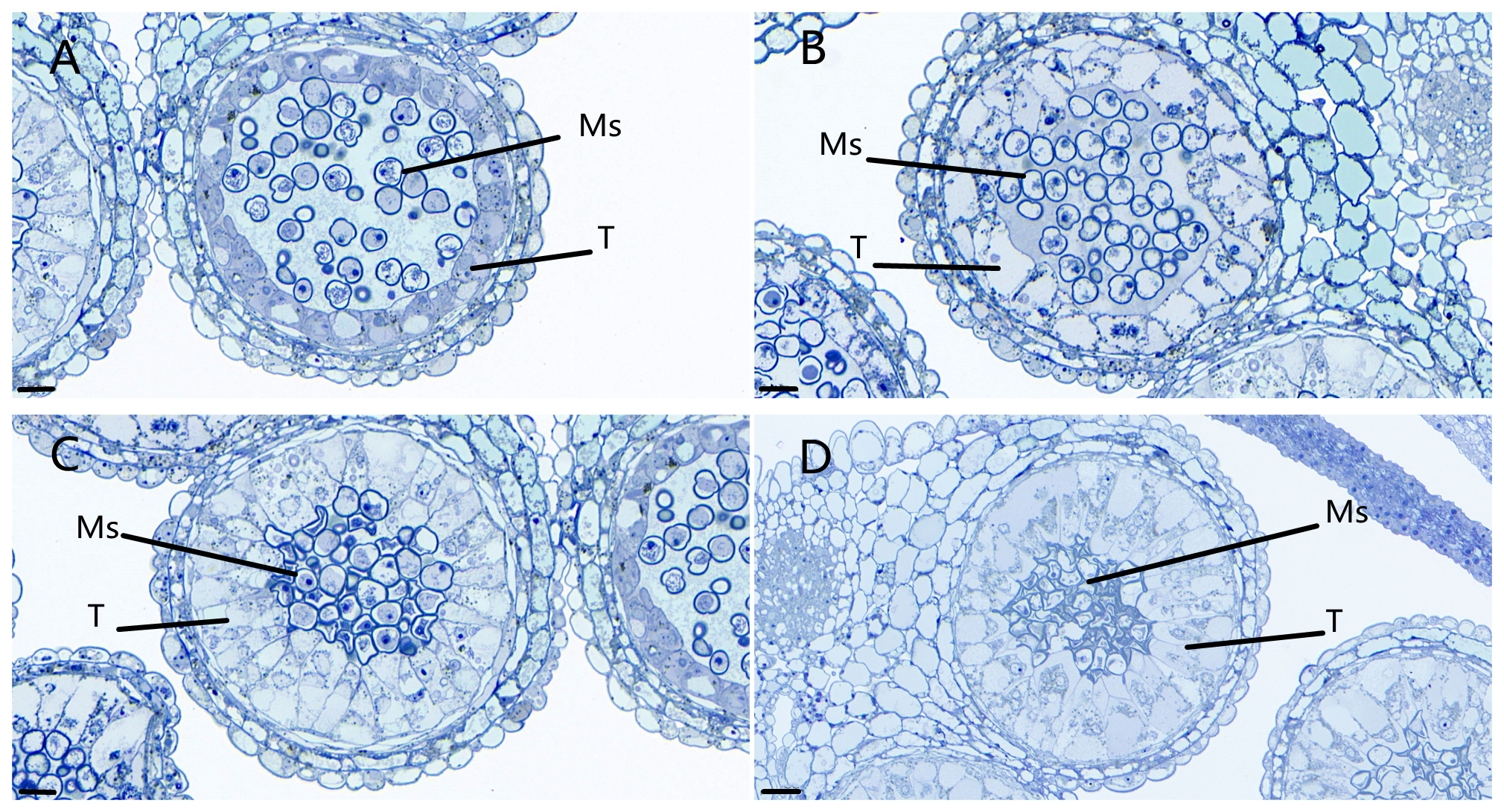

Supplement: Supplementary file 1 [file ijms-23-02004-s001.zip › Supplementary Figure S1.jpg]

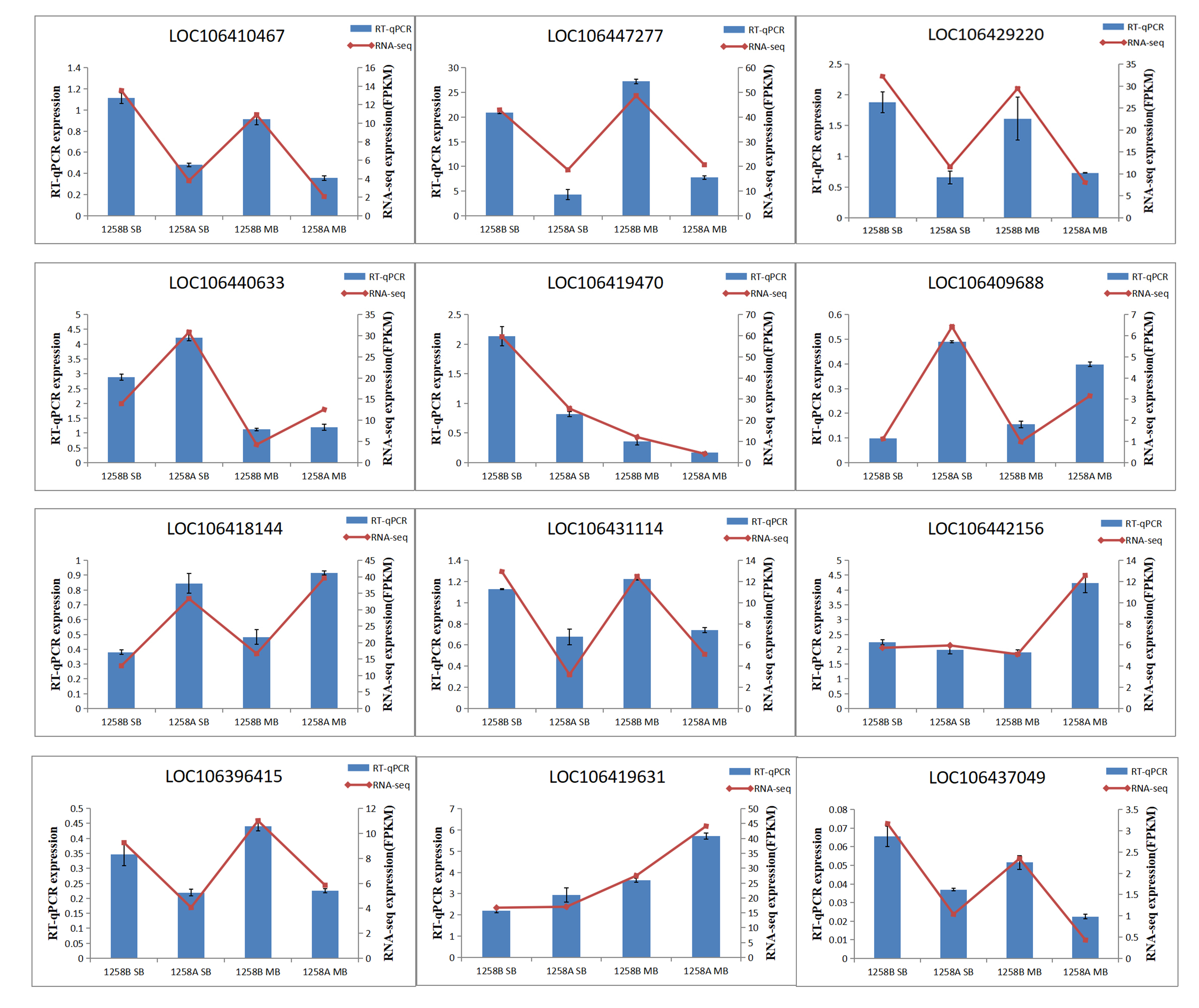

Supplement: Supplementary file 1 [file ijms-23-02004-s001.zip › Supplementary Figure S2.jpg]
